# Supplementary material for: Broadband visual stimuli improve neuronal representation and sensory perception
Source: Nat Commun. 2025 Mar 26;16:2957. doi: 10.1038/s41467-025-58003-1 (PMC11947450; doi:10.1038/s41467-025-58003-1)
Supplement: Supplementary file 1 — Supplementary Information [file 41467_2025_58003_MOESM1_ESM.pdf]

Broadband visual stimuli improve neuronal representation and sensory perception

Elisabeta Balla, Gerion Nabbefeld, Christopher Wiesbrock, Jenice Linde, Severin Graff, Simon Musall, Björn M. Kampa

Supplementary Figures

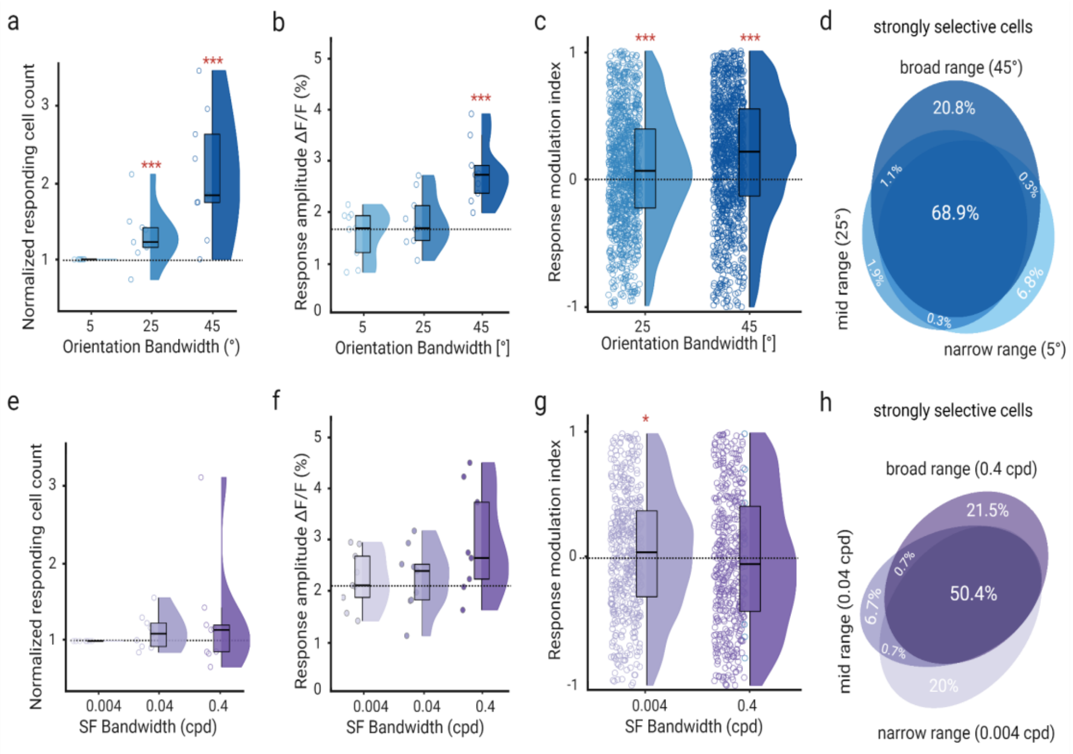

Figure S1. Differences between orientation bandwidths are consistent across animals.
(**a**) Counts of responsive neurons to each orientation bandwidth, normalized by the number of cells responding to narrow (n = 9 mice). (**b**) Responses amplitudes for all neurons with a significant response for at least one stimulus (2096 responsive neurons out of 4892 neurons in total). (**c**) Response modulation index (RMI) for neurons with significant positive responses to any enriched stimulus (RMI = (enriched – narrow)/ (enriched + narrow)). Mid band modulated neurons: 57% positively (RMI = 0.39 ± 0.01, mean ± s.e.m.), 43% negatively (RMI = -0.33 ± 0.01, mean ± s.e.m). Broadband modulated neurons: 67% positively (0.20 ± 0.01, mean ± s.e.m), 33% negatively (-0.18 ± 0.01, mean ± s.e.m.). (**d**) Distribution of neurons that selectively respond to a single or multiple orientation bandwidth stimuli or common responders that significantly responded to all stimulus bandwidths. The percentage of neurons from all neurons that significantly and selectively respond to either of the bandwidth stimuli is shown (366 neurons from 18 sessions in 9 mice). (**e**) Same as a) for spatial frequency bandwidth. (**f**) Same as b) for spatial frequency bandwidth (n = 9 mice, 888 responsive out of 3185 total neurons). (**g**) Same as c) for spatial frequency bandwidth. Mid band modulated neurons: 54% positively (0.40 ± 0.02, mean ± s.e.m), 46% negatively (-0.37 ± 0.02, mean ± s.e.m). Broadband modulated neurons: 48% positively (0.30 ± 0.02, mean ± s.e.m), 52% negatively (-0.18 ± 0.02, mean ± s.e.m). (**h**) Same as d) for spatial frequency bandwidth stimuli (135 neurons from 16 sessions in 9 mice). For visualization only, outliers were excluded from distributions in panels e-h. Box plots indicate the median (horizonal line), interquartile range (box bounds: 25th–75th percentiles), and whiskers (1.5× interquartile range). In panels a, b, e, and f, stars show significance for the Wilcoxon signed rank test against narrow responses, dotted line is narrow median. In panels c and g stars show significance for an LME model test against zero, dotted line is zero. The significance threshold was adjusted upon Bonferroni correction for number of tests performed (α = 0.025, 2 tests, panels a-c, e-g).

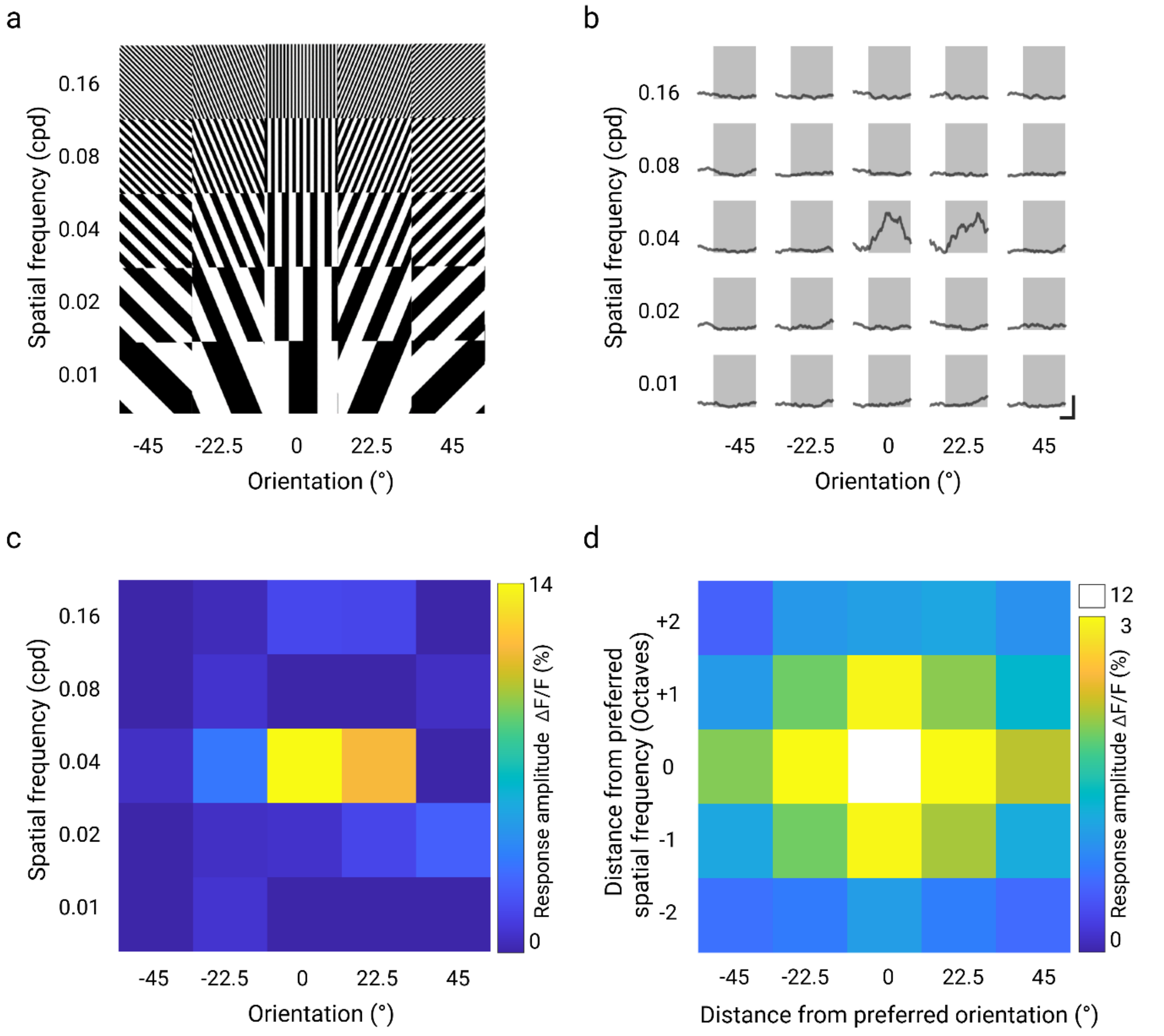

Figure S2. V1 tuning specificity to orientation and spatial frequency of grating stimuli.
(**a**) Overview of 25 combinations of visual grating stimuli, consisting of 5 different orientations (-45°; -22.5°; 0°; 22.5°; 45°) and spatial frequencies (0.01 cpd; 0.02 cpd; 0.04 cpd; 0.08 cpd; 0.16 cpd). All stimuli were presented as pseudorandom sequences to 3 awake mice in 6 sessions in total. (**b**) Mean response traces of an example V1 neuron for each stimulus combination. The shaded area is the stimulus duration. (Scale bar: 1s, 20% ΔF/F). (**c**) The average response strength to each stimulus combination for the neuron is shown in panel b. (n = 20 presentations per stimulus). Visible is a narrow orientation and spatial frequency tuning. (**d**) Averaged tuning specificity for all responsive V1 neurons (n = 971 cells). The response matrix was shifted for each neuron so that the peak orientation and spatial frequency responses were in the center. Datapoints that were not measured because they were outside of our tested range were set to NaNs and not weighted. X- and Y-axes, therefore, show the distance from the preferred orientation and spatial frequency to measure the width of the population orientation and spatial frequency tuning within the presented stimulus range. Paired Wilcoxon signed rank test of the full-width half maximum (FWHM) of a Gaussian fit for the central X-axis (for orientation) and Y-axis (for spatial frequency) showed a small but significant difference between the two tuning widths: $FWHM_{orientation}$ = 1.76 ± 0.04 bins, $FWHM_{spatial\ frequency}$ = 1.67 ± 0.04 bins (Wilcoxon signed rank test, p = 1.91 x 10-38). This suggests that V1 neurons were slightly less selective for different grating orientations. Importantly, this is the opposite of what would have been expected if the selective increase in neural responses to broad orientation bandwidth had been solely caused by additional recruitment of V1 subpopulations with very narrow orientation tuning.

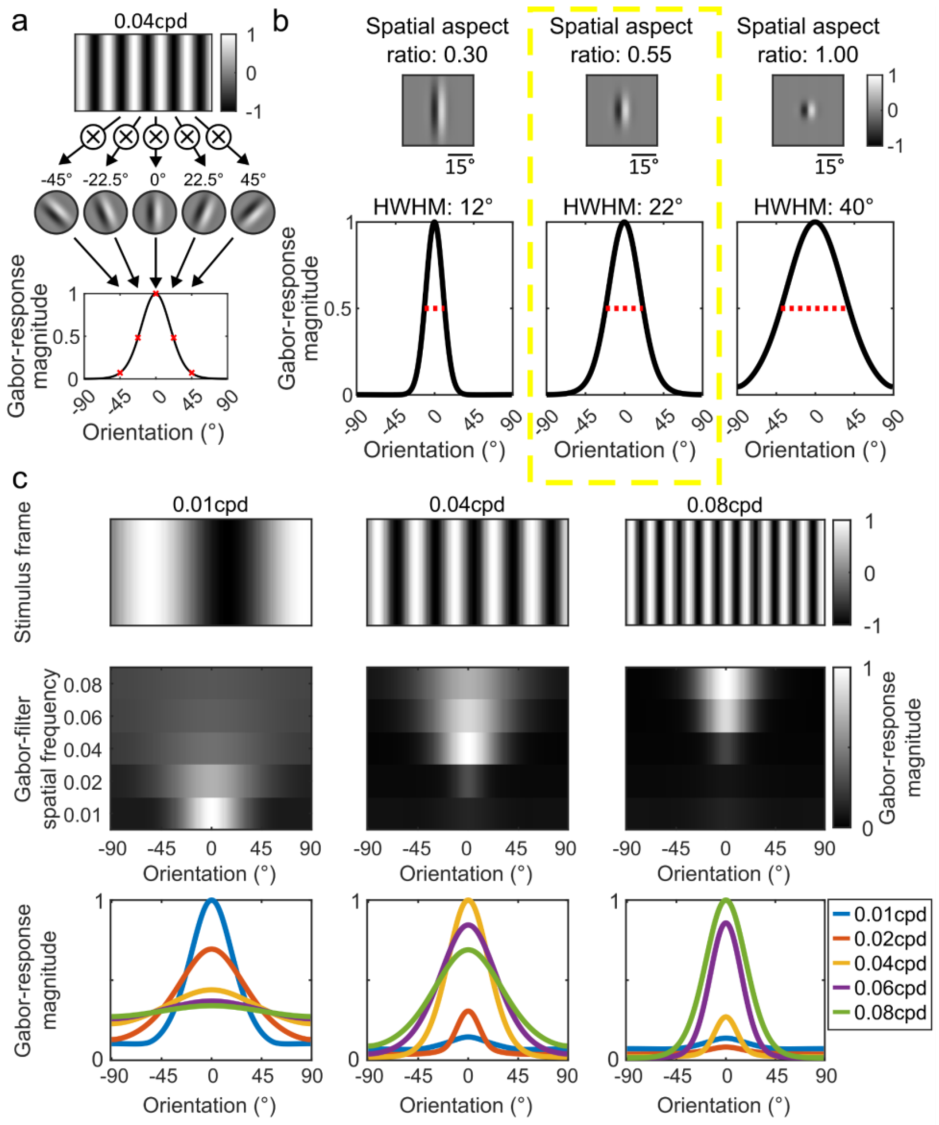

Figure S3. A bank of Gabor filters to model mouse V1 neuron responses.
(**a**) Schematic of Gabor filter responses. Top: Example image of a sine wave grating stimulus with a spatial frequency of 0.04 cpd. Middle: different orientations of Gabor filters with a spatial frequency of 0.04 cpd and a spatial aspect ratio of 0.55, visualized for an example phase of the complex spatial kernel. Bottom: grating response curve over Gabor filters with different orientations. (**b**) The spatial aspect ratio of the used Gabor filters was adjusted to match the orientation tuning of mouse V1 neurons in response to the same 0.04cpd grating shown in a). Top row: visualization of the spatial filter kernels with different aspect ratios. The scale bar indicates the size of each Gabor filter in visual angles relative to stimulus space. Bottom row: Normalized response magnitude across different orientations of the Gabor filter with the corresponding spatial aspect ratio (title in the top row). The red line indicates the half-maximum response at which the half-width half-maximum (HWHM in the panel titles) of the resulting orientation tuning curve was calculated. The yellow box indicates the spatial aspect ratio that was used for all the following model fits. (**c**) Normalized responses of Gabor filters with different spatial frequencies and orientations to different grating stimuli. To represent the spatial frequency tuning of V1 neurons, Gabor filters for five different spatial frequencies (0.01 cpd, 0.02 cpd, 0.04 cpd, 0.06 cpd and 0.08 cpd) were used to model V1 responses. Responses for each of the Gabor filters were normalized to their peak response to a sine wave grating that matched their corresponding spatial frequency. Top row: 3 example sine wave grating stimuli with different spatial frequencies, indicated in the panel titles. Middle rows: Response magnitudes to the stimulus shown above across different orientations of the five types of Gabor filters with different spatial frequency tuning. Gabor filters displayed the strongest responses to stimuli that matched their spatial frequency tuning. Bottom: Same as middle panels but showing the tuning curves for each Gabor filter type individually for better comparability across spatial frequency tuning of the different Gabor filters.

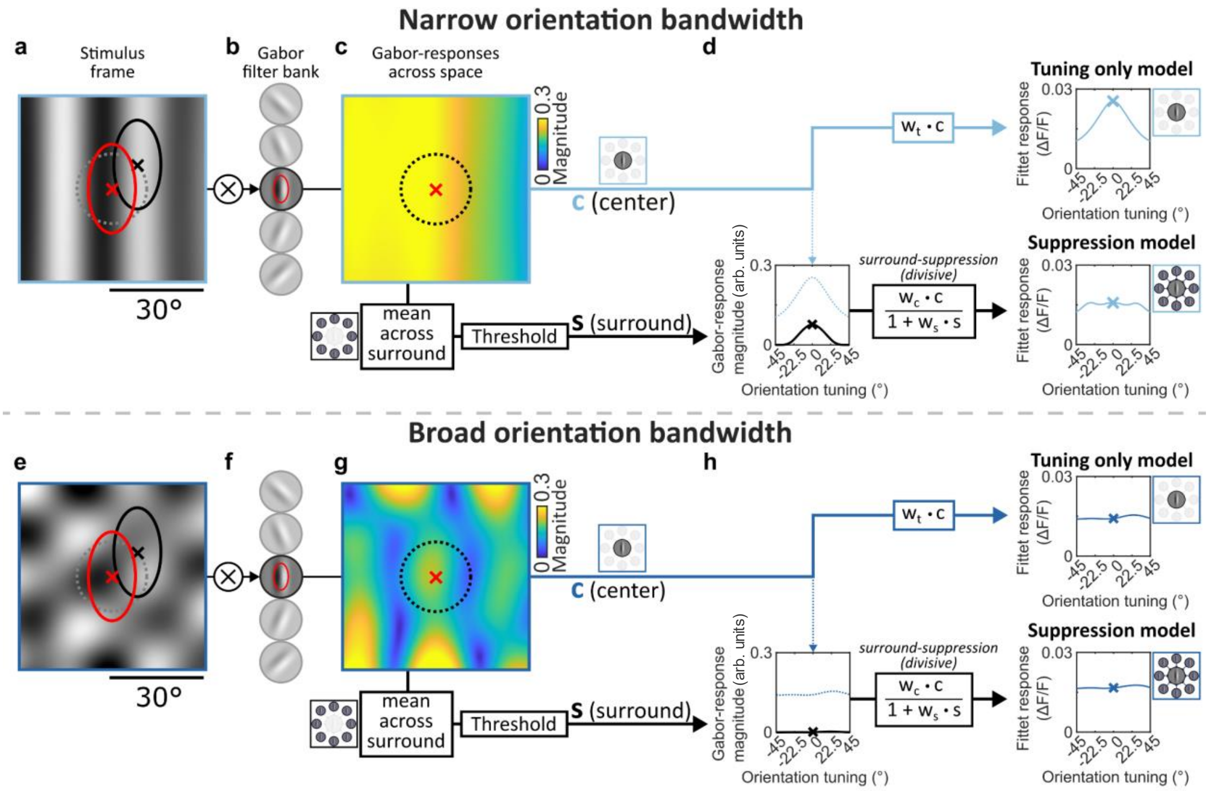

Figure S4. A Gabor filter model to fit V1 responses to narrow and broad orientation bandwidth stimuli. (**a**) Example narrow-band frame. Red ellipse and cross indicate the outline and midpoint of the center Gabor, respectively. Black ellipse and cross indicate the location of an example Gabor filter, contributing to the surround suppression in the Suppression model. Dashed circle indicates the midpoint locations of other surround Gabor filters (radius = ~11.7°, matching the dashed line in c). (**b**) Bank of Gabor filters, comprising filters with five orientations between -45° and 45° and five different spatial frequencies (0.01, 0.02, 0.04, 0.06, 0.08 cpd). Highlighted is an example Gabor filter kernel with 0° and 0.04 cpd tuning. Red ellipse shows the half-maximum of the Gabor envelope, similar to a) (**c**) Response magnitudes of Gabor responses across space after applying the example filter of stimulus frame. Each pixel indicates the response of this Gabor at different locations in the frame (mapped in correspondence to the Gabor filter midpoint). From these responses across space, either only the center Gabor response (red cross; Tuning only model) or both the center and the surrounding responses were used (dashed circle; Suppression model). (**d**) Tuning-only model, predicts neuronal responses to the center by fitting scaling factor w_t, which scales average Gabor response magnitudes to average V1 responses (see Figure 3b, c). Tuning-only model, therefore, presents the best fit when describing neural responses as simple Gabor filters with different orientation and spatial frequency tuning, averaged over all stimulus frames of the corresponding condition. Suppression model extracts Gabor responses of the same orientation that spatially surround the center Gabor. Surround magnitudes are then averaged and thresholded with a fitted offset to obtain the surround response. The surround region limits, thresholding offset, and the center and surround weights (w_c, w_s) are simultaneously fitted and divided the center by the surround response. Tuning curve markers highlights the link to the 0° Gabor shown in (b) and (c). (**e-h**) Same models were applied to broadband stimuli and fitted simultaneously to the measured neural responses to narrow and broadband stimuli for fit optimization (see Figure 3b, c). Panels c, d, g, and h were created in BioRender. Balla, E. https://BioRender.com/g05l789 (2025).

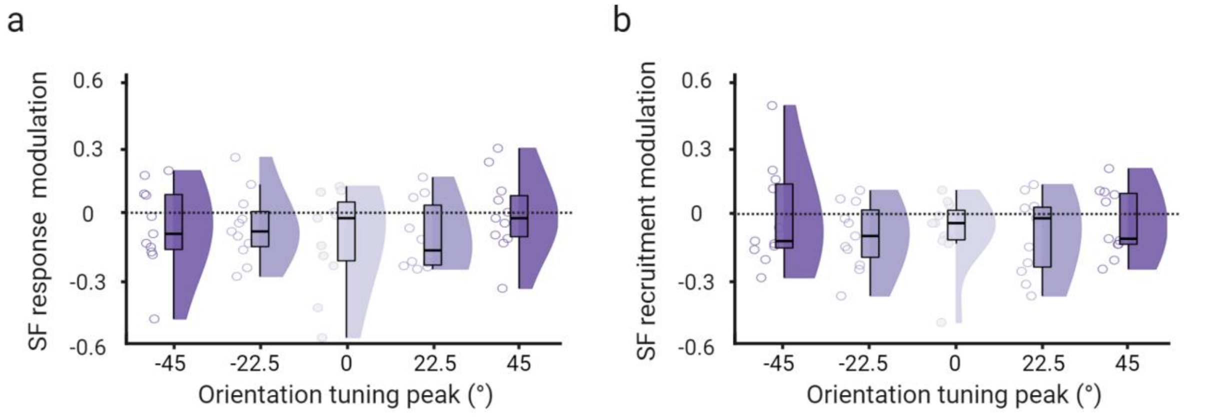

Figure S5. SF response modulation is independent of neural orientation tuning.
(**a**) Mean response modulation of orientation-tuned cells by expanding the spatial frequency bandwidth (n = 11 sessions in 4 mice). Neurons in each session were binned based on their orientation tuning within the range of the broad orientation bandwidth stimulus. The horizontal black dotted line at 0 indicates no bandwidth modulation. As in our earlier recordings (Fig. 2), expanding the spatial frequency bandwidth did not have an impact on neuronal response amplitudes, irrespective of their orientation tuning (Spatial frequency response modulation index (SF RMI): SF RMI-45° = -0.06 ± 0.06, T = -1.37, p = 0.19; SF RMI-22.5° = -0.06 ± 0.05, T = -1.38, p = 0.18; SF RMI0° = -0.12 ± 0.07, T = -1,83, p = 0.08; SF RMI22.5° = -0.03 ± 0.09, T = -0.41, p = 0.68; SF RMI45° = -0.01 ± -0.06, T = -0.17, p = 0.87, mean ± s.e.m., LME model test against zero, n = 11 recordings from 5 mice). (**b**) Same as panel a) but for recruitment modulation of orientation-tuned cells by expanding the spatial frequency bandwidth. The SF recruitment modulation index was calculated as the difference between the numbers of responsive neurons to narrow and broad SF bandwidth stimuli divided by their sum. We also found no changes in the recruitment of visually-responsive neurons or a clear dependence on their orientation tuning in panel b. Box plots indicate the median (horizonal line), interquartile range (box bounds: 25th–75th percentiles), and whiskers (1.5× interquartile range). Stars in all panels mark significant differences against 0 based on an LME model. The significance threshold was α = 0.01 after Bonferroni correction for performing five tests.

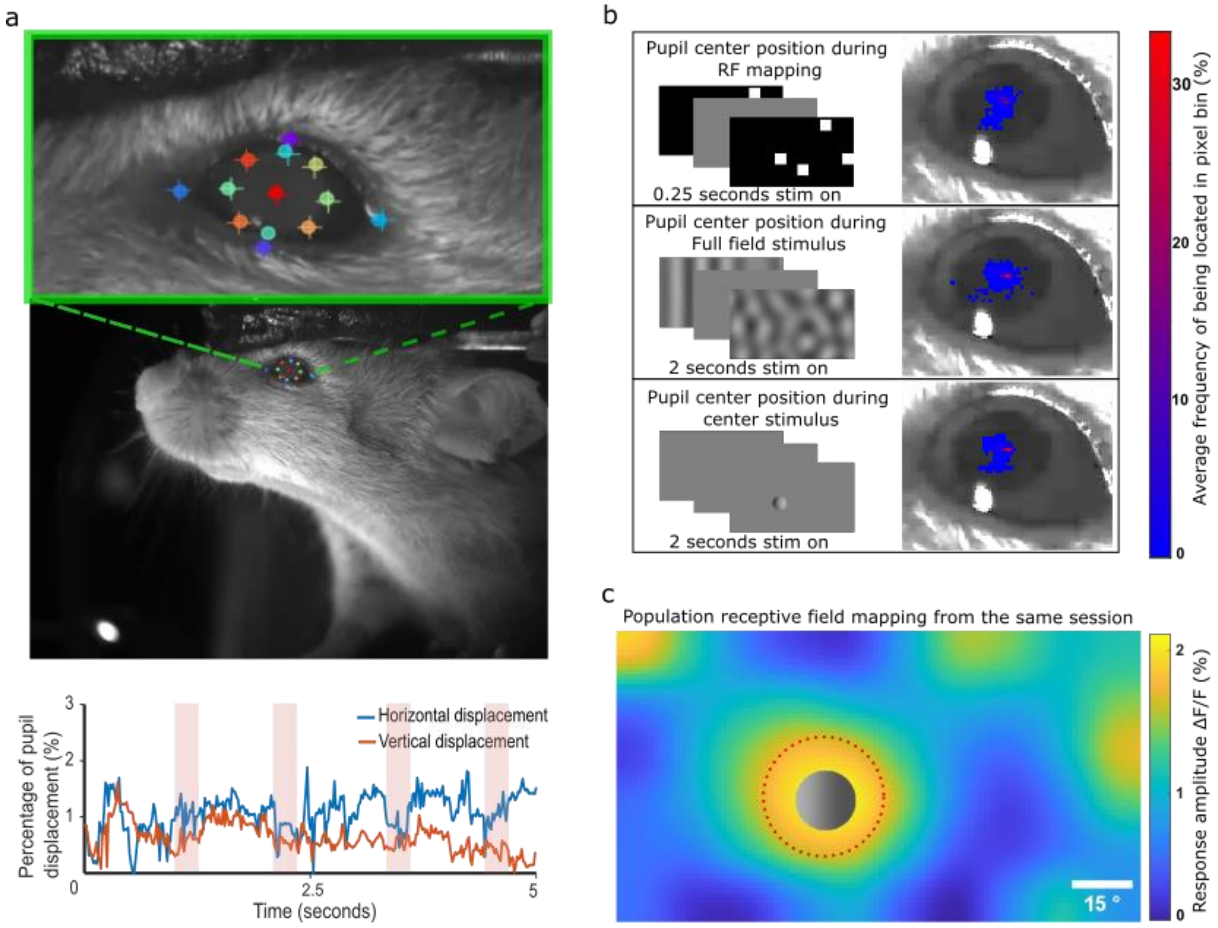

Figure S6. Pupil movements do not affect neural responses to the stimulus in the receptive field center. (**a**) Example frame from the behavior camera. Expanded on top is the mouse eye, labeled at several locations to train a DeepLabCut[1] model that followed the eye position throughout the session. We labeled the top, bottom, left, and right eyelid edges to obtain a reference of the outer range to where the pupil could move. Then, we tracked the pupil location (eight reference points around the pupil edge and one at the center). The bottom panel shows example pupil movements during a randomly selected 5-second time window during the receptive field (RF) mapping. The pupil displacement is shown in pixels, normalized by the total possible displacement range for each axis (horizontal or vertical). The pink shaded areas mark the sparse noise stimulus presentations that were used for RF mapping (0.25 seconds stimulus duration with 1-second inter-stimulus intervals on a mean gray screen). (**b**) Mean heatmap of the center pupil locations during either the RF mapping (top), full-field stimulus presentations (middle), and the center stimulus presentations (bottom). For visualization purposes, we used 2x2 pixel binning of pupil positions relative to the camera resolution to calculate the fraction of frames containing the pupil center in each pixel bin ("location frequency"). This was done separately for each stimulus presentation condition (all RF stimuli, all full-field stimuli, or all center stimuli). For each condition, we found that the pupil remained largely invariant at the eye center with a very restricted surrounding area with low position frequency. This shows that changes in pupil position are not a major confound for interpreting neural responses during the different stimulation paradigms. (**c**) RF mapping of the center responding cells from the same example session. The RF map was matched to the screen size, and an example center stimulus is shown at the center of the RF center on the screen. The scale bar is 15°. Average RF size (dotted red circle surrounding the stimulus) of the population across all the sessions was 30.45° ± 2.04° (mean ± SD), ensuring that the center stimulus was placed within the receptive field of the "center responding" cells, shown in Fig. 4.

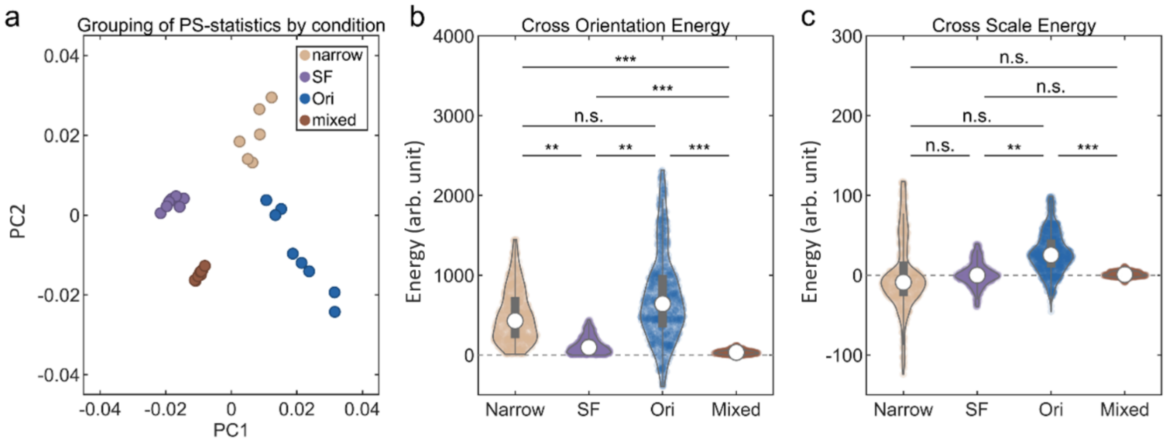

Figure S7. Stimulus separability and coefficient magnitude statistics.
(**a**) Overview of overall variability and separability among the ten renderings for each stimulus condition ('Narrow' - beige, 'SF' - purple, 'ORI' - blue, 'Mixed'- brown). Texture statistics for each stimulus frame was calculated using the Portilla and Simoncelli analysis ('PS-statistics', see also Methods: 'Stimulus predictability' and 'Stimulus separability')[2]. Then, a principal component analysis across all PS-statistics (including pixel statistics, auto-correlations, magnitude statistics, as well as statistics of the low- and high-frequency residuals) was computed and averaged across all frames of a rendering. Projection onto the first two principal components (PC) is shown for individual renderings. Data points spread shows the variability in texture statistics between renderings of the same stimulus condition. Largest variability is seen in narrow and ORI conditions. However, individual stimulus renderings formed non-overlapping clusters, demonstrating that they contain distinct combinations of PS-statistics that are unambiguously distinguishable between stimulus categories. (**b**) The effect of expanding spatial frequency or orientation bandwidth on complex structures or second-order textures was examined by comparing coefficient magnitude statistics. Cross Orientation Energies, derived from PS-statistics, were computed from energy correlations between the 0° and the ±45° sub-band magnitudes at the coarsest spatial scale of the steerable image pyramid decomposition. Expanding spatial frequency bandwidth, especially in "Mixed", reduced cross-orientation energy, while orientation bandwidth had no significant effect. This suggests that expanding spatial frequency bandwidth reduces image structures (e.g., edges, bars, corners) on the coarsest spatial scale. (**c**) Comparison of cross-scale energies between stimulus conditions. Shown are average cross-correlation of 0° filter sub-band magnitudes between the two coarsest spatial scales of the image pyramid. Expanding orientation bandwidth resulted in significantly higher cross-scale energies compared to SF and Mixed. However, neither expanding spatial frequency- nor orientation bandwidth significantly changed cross-scale energies compared to narrow stimuli, suggesting that there are no strong and consistent changes in cross-scale energy. Box plots indicate the median (horizonal line), interquartile range (box bounds: 25th–75th percentiles), and whiskers (1.5× interquartile range). Significance was tested with a Wilcoxon–Mann–Whitney U test with Bonferroni-correction ($\alpha = 0.0167$, 3 tests) comparing the cross energies of individual stimulus realizations, averaged over individual frames.

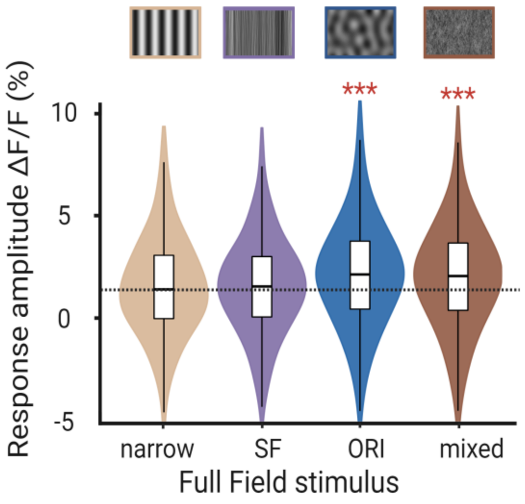

Figure S8. Increased neural response amplitude to the full-field mixed broadband condition. Difference between mean response amplitude versus baseline for each full-field stimulus category. Shown are all neurons with a significant response to either of the full-field stimuli (2959 responsive neurons out of 8210 neurons in total). The black dotted line shows the median for the narrow stimulus. Response amplitudes: narrow = 3.37% ± 0.19%; SF =3.30% ± 0.18%, Wilcoxon signed rank against narrow full-field response, $p = 0.98$; ORI = 3.95% ± 0.20%, $p = 8 \times 10^{-31}$; mixed = 3.90% ± 0.23%, $p = 1.01 \times 10^{-8}$, mean ± s.e.m., $n = 2959$ neurons from 5 mice. For visualization only, outliers were excluded from distributions. Box plots indicate the median (horizonal line), interquartile range (box bounds: 25th–75th percentiles), and whiskers (1.5× interquartile range). The significance threshold was $\alpha = 0.0167$ after Bonferroni correction for performing three tests.

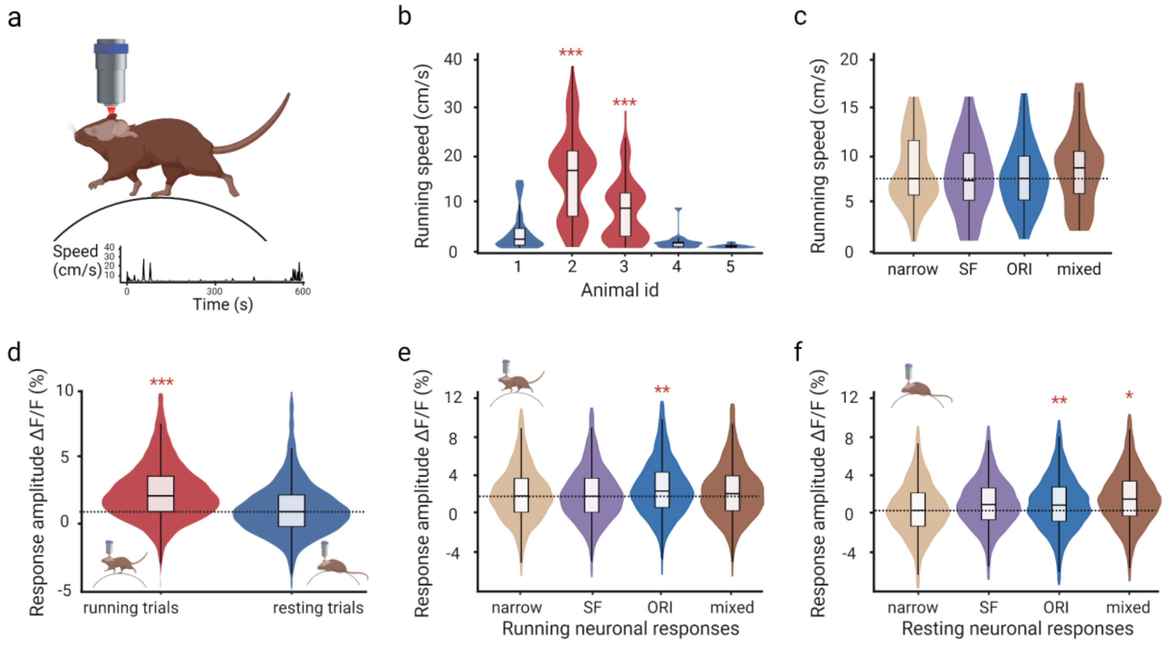

Figure S9. Broadband orientation stimuli increase neural responses during different behavioral states. (**a**) Illustration of behavioral data acquisition during 2-photon imaging. Wheel movements were collected via a rotary encoder (bottom trace). (**b**) The average running speed varied across individual animals and was roughly split between consistent runners (animals 2 and 3) and rather passive observers (animals 1, 4, and 5, n = 600 trials per mouse, 5 mice, Wilcoxon ranked sum test against the median across all mice). (**c**) Average running speed across all presentations of each stimulus condition for all mice. There were no significant differences in running speed across conditions. Dotted line is median of the narrow stimulus average running speed (n = 55 sessions per stimulus across 5 mice, Wilcoxon ranked sum test against the other stimuli). (**d**) Running increases visual response amplitude. Shown are average response amplitudes to all visual stimuli when mice were either resting or running on the wheel. Black dotted line is the median response amplitude of resting trials. Responses to visual stimulation were significantly larger during running compared to resting trials (n = 5043 responding cells, 5 mice, LME model comparison). (**e**) Neural responses are increased for broad orientation bandwidth during running trials. Shown are the average response amplitude across all running trials for all neurons in each stimulus condition. Dotted line is median of the narrow stimulus response amplitude (n = 4541 responding cells, 5 mice, LME model testing against narrow). (**f**) Neural responses are increased for broad orientation bandwidth during resting trials. Shown are average response amplitudes across all resting trials for all neurons in each stimulus condition. Dotted line is median of the narrow stimulus response amplitude (n = 4322 responding cells, 5 mice, LME model testing against narrow). For visualization only, outliers were excluded from distributions in panels b-h. Box plots indicate the median (horizonal line), interquartile range (box bounds: 25th–75th percentiles), and whiskers (1.5× interquartile range). The significance threshold was adjusted after Bonferroni correction for performing the pertaining number of tests ($\alpha = 0.01$, 5 tests, panel b; $\alpha = 0.0167$, 3 tests, panels c, e, f). Panel a was created in BioRender. Balla, E. https://BioRender.com/g05l789 (2025).

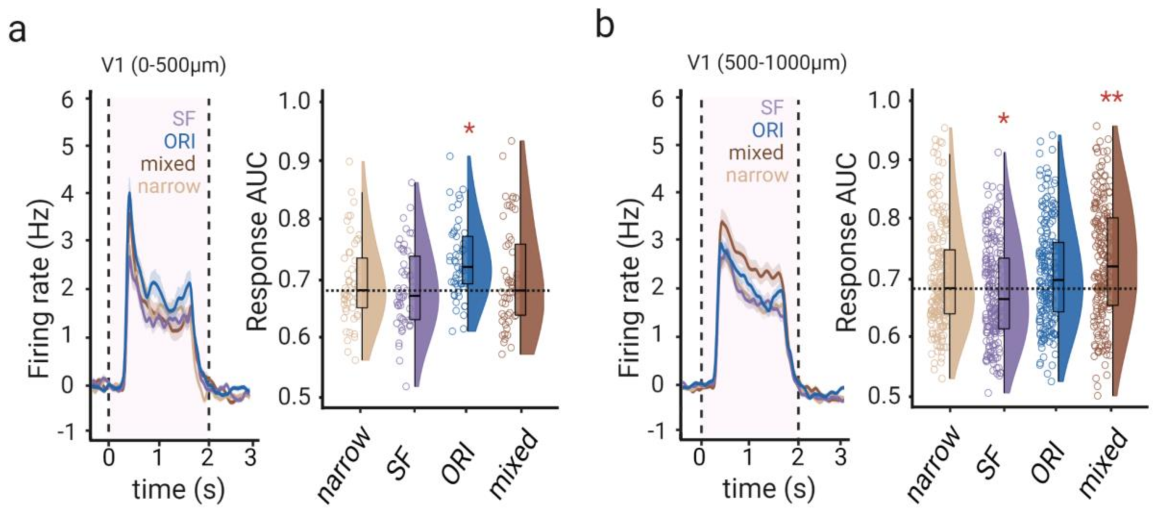

Figure S10. Differences in broadband response tuning between superficial and deeper V1 neurons.
(**a**) Left: Mean firing rates of superficial (0-500 µm deep) neurons in V1. The shaded area around the solid lines shows the s.e.m. The area between the vertical dashed lines is the stimulus presentation window (n = 53 responding units from 3 animals). Right: Response AUCs against baseline for each stimulus condition. (**b**) Same as in panel a but for deeper V1 neurons between 600-1000 µm (n = 200 responding units from 3 animals). For visualization only, outliers were excluded from distributions in the panels. Box plots indicate the median (horizonal line), interquartile range (box bounds: 25th–75th percentiles), and whiskers (1.5× interquartile range). Stars in all panels mark significant differences from the LME model test against narrow. The significance threshold was α = 0.0167 after Bonferroni correction for performing three tests.

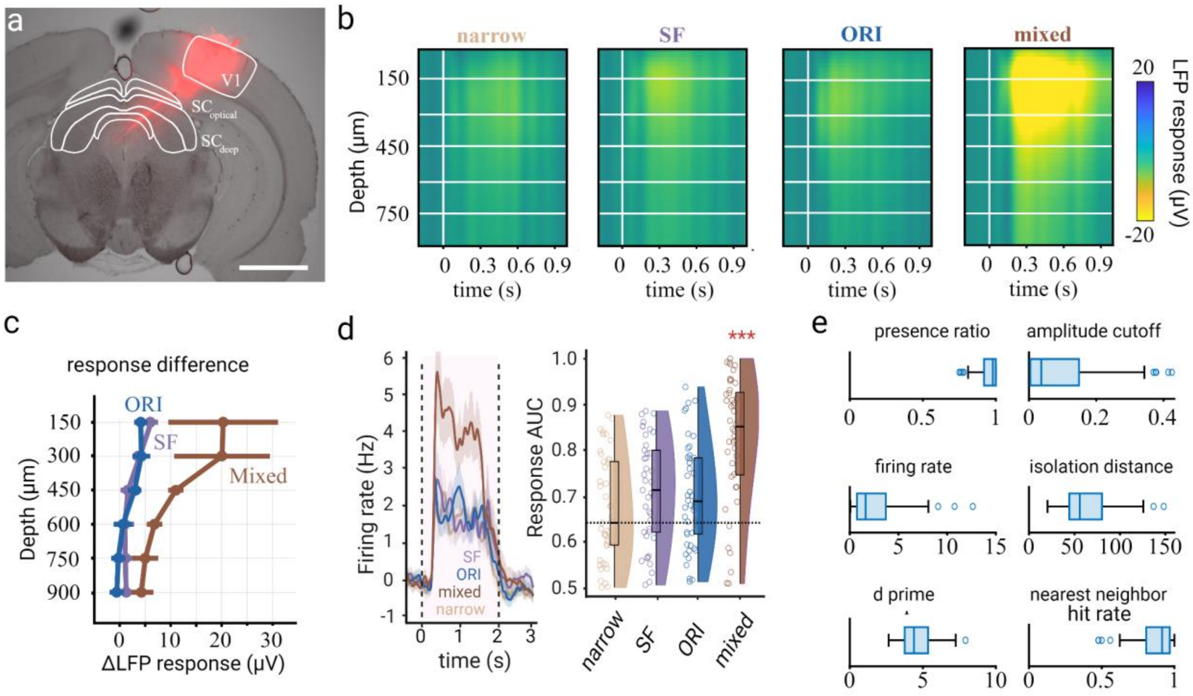

Figure S11. Neurons in the optical SC preferentially respond to the mixed broadband stimulus condition. (**a**) Example brain slice with electrode tract, following the electrophysiological recordings. The fluorescent DiD signal from the Neuropixels probe is visible in pink, originating in V1 and reaching different SC layers. The scale bar is 1.5 mm. Brain region annotations are based on the Allen Mouse Brain Common Coordinate Framework (CCF)[3]. (**b**) Electrophysiological LFP responses to each stimulus condition at different depths of the SC. Different depths from the SC surface are marked by white lines. The SC surface was determined by the first channel with observable visual responses in the LFP, which occurred at a recording depth of 1.63±0.16 mm across six recordings from 3 mice. Colors show voltage changes after stimulus onset relative to baseline. (**c**) Differences in LFP responses for each stimulus condition (SF: purple; ORI: dark blue; mixed: brown) compared to the LFP response to the narrow stimulus across depths. Error bars show mean and s.e.m. (n = 6 recordings from 3 mice). Neural responses were strongest for the mixed condition in the superficial SC layers, and responses to all stimulus conditions were lower in deeper SC layers. (**d**) Left panel: Mean firing rate of SC neurons. The shaded area around the solid lines shows the s.e.m. The area between the vertical dashed lines is the stimulus presentation window (n = 46 responding units from 3 animals). Right panel: Response AUCs for different stimulus conditions for individual neurons. Stars mark significant differences from the LME model test against the narrow condition. The significance threshold was α = 0.0167 after Bonferroni correction for performing three tests. For visualization only, outliers were excluded. Box plots indicate the median (horizonal line), interquartile range (box bounds: 25th–75th percentiles), and whiskers (1.5× interquartile range). (**e**) Spike sorting quality metrics for all manually selected good clusters across all Neuropixels recordings. The quality metric results are consistent with well-isolated units, such as a high presence ratio, d-prime, and nearest-neighbor hit rate, with a low amplitude cutoff.

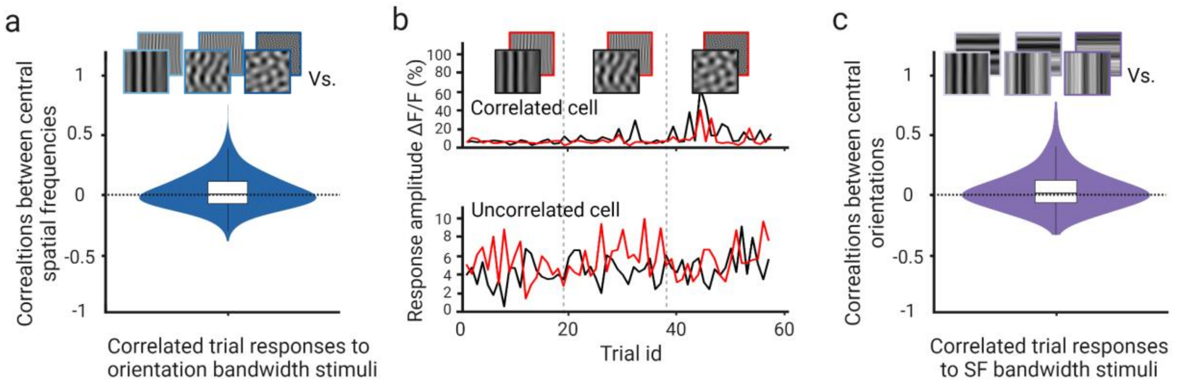

Figure S12. Bandwidth selectivity differs across central spatial frequencies or orientations.
(**a**) Neuronal response correlations were analyzed for stimuli with different orientation bandwidths and either low (0.04 cpd) or high (0.16 cpd) central spatial frequency (SF). Stimulus examples shown on top. Responses from neurons significantly activated by any bandwidth (19 trials per bandwidth, 57 responses in total) were correlated across SF conditions. No significant correlation was found (r = 0.02 ± 0.003, mean ± s.e.m., n = 2097 neurons, Wilcoxon test, p = 0.073), indicating no systematic preference for a specific bandwidth independent of SF. Additionally, bandwidth preference showed little overlap across SFs: 9% of narrow-preferring neurons at 0.04 cpd remained narrow-preferring at 0.16 cpd, 5% for mid-preferring, and 13% for broad-preferring. Note that the overlap of bandwidth-selective neurons across the central SFs was not a result of the correlation analysis but is mentioned here for completeness. (**b**) Response amplitudes to all stimuli for two example cells from a), stimuli shown above. Top cell showed a high response correlation and preferentially responded to broad orientation bandwidth stimuli with either central SF. Dotted lines mark the last trial for each bandwidth condition. Red lines - high central SF responses, black lines - low central SF responses. Bottom example shows a de-correlated neuron response vector. (**c**) Same as a) for correlations to stimuli with different SF bandwidths and a central orientation of either 0° or 90°. Lack of significant difference from 0 suggests that SF bandwidth selectivity of neural responses is not independent of the central orientation (Pearson correlations: r_{value} = 0.04 ± 0.005, mean ± s.e.m., n = 888 neurons, Wilcoxon signed-rank test against 0, p = 0.069). Again, little overlap between the SF bandwidth selective neurons across the two central orientations: 8% of narrow SF bandwidth preferring neurons at 0° central orientation were also narrow SF bandwidth preferring at 90° central orientation, 3% of mid preferring at 0° were also mid preferring at 90° and 6% of broad preferring at 0° were also broad preferring at 90°. Box plots indicate the median (horizonal line), interquartile range (box bounds: 25th–75th percentiles), and whiskers (1.5× interquartile range).

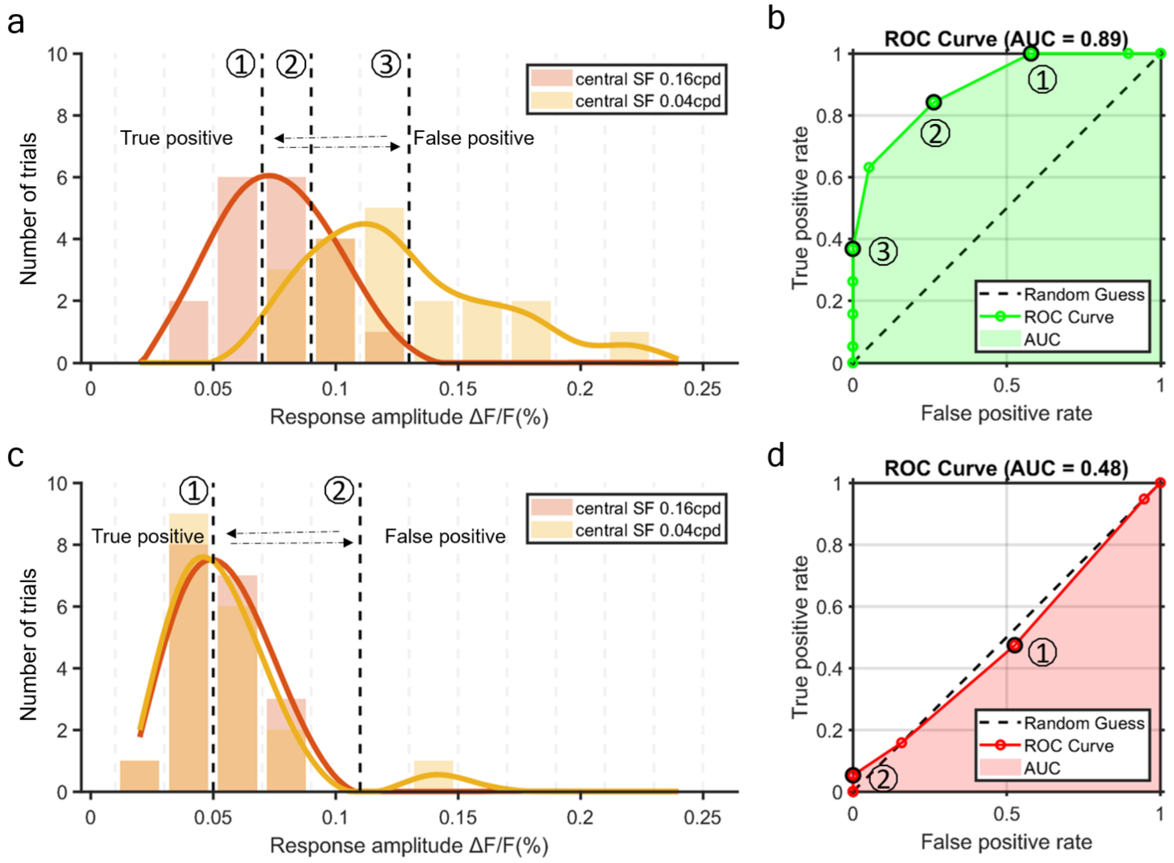

Figure S13. Example central SF response distributions and corresponding ROC curves for a strong central SF discriminator neuron (top row) and a weak central SF discriminator neuron (bottom row).

(**a**) Histogram of response amplitude distributions for the broad orientation stimulus condition (45° orientation bandwidth) with a low central SF (0.04 cpd, light orange, n = 19 trials) versus a high central SF (0.16 cpd, dark orange, n = 19 trials) for an example strong central SF discriminator neuron. Light and dark orange distribution overlap minimally (Wilcoxon ranked-sum test, p = 0.1 x 10^{-4}). (**b**) Receiver-operator characteristic (ROC) curve showing true vs. false positive rates for distributions in panel a. When response distributions are distinct at a given threshold, the true positive rate is high, and the false positive rate is low, moving the ROC curve away from chance (dotted line). Varying the threshold shifts ROC values, with AUC quantifying separability (0.5 = no discrimination, 0/1 = perfect discrimination). For responses to central spatial frequencies and to central orientations (Fig. 6), we used the absolute AUC (AUCabs = |AUC-0.5|*2) as a measure of discriminability. AUC_{abs} is bound between 0 and 1, with larger values indicating increased discriminability of neural responses between the two stimulus conditions. Black circles (1-3) on the ROC curve correspond to thresholds in panel a. The more rightward the threshold is placed, the more the corresponding ROC marker drifts to the left, decreasing the false positive rate but also the true positive rate at some point. In position 1, the true positive rate is 1 since all low central SF responses are right of the threshold. However, 58% of high central SF responses are also right, giving a false positive rate of 0.58. Moving the threshold to position 3 reduces false positives to 0 but also lowers the true positive rate. An optimal separating point would be the threshold in position 2, where the best ratio between true positive (0.84) and false positive (0.26) rates is found. (**c**) Same a) for a non-discriminating neuron (see the large overlap between the distributions, Wilcoxon ranked-sum test, p = 0.81). (**d**) Same as b), for the non-discriminating neuron. Here, the orange distributions largely overlap, and true and false positive rates are symmetric, regardless of where the threshold is placed.

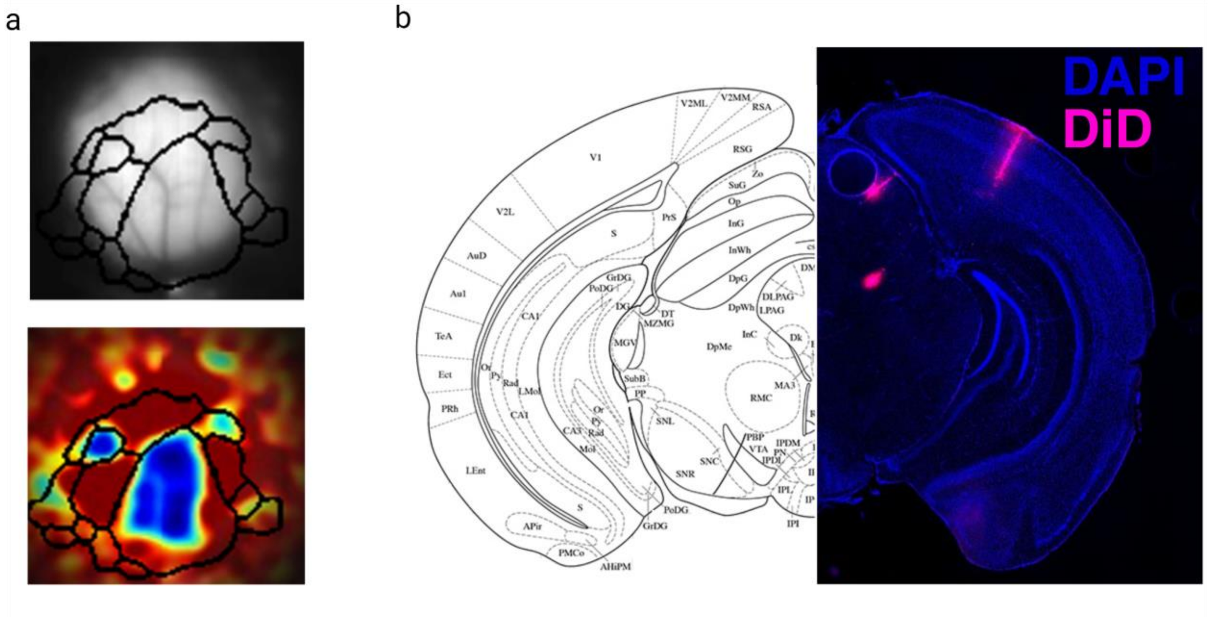

Figure S14. Retinotopic identification of visual areas and histology for Neuropixels recordings.
(**a**) Example retinotopic mapping result from a mouse in which we performed Neuropixels recordings. The inferred border areas are drawn on top of the vessel architecture (top) based on the visual sign map that informs visual area locations (bottom). Recordings were done in the center of V1 (large blue area in the bottom panel). (**b**) Example histology results following the electrophysiological recordings. Fluorescent marks toward the midline are from recordings where the probe reached the SC. The fluorescent trace in the cortex is from a recording where the probe was placed perpendicular to the cortical surface. Blue is DAPI staining, and pink is the DiD trace of the Neuropixels probe insertion. The opposite hemisphere is replaced by a scheme of the mouse brain atlas (The mouse brain in stereotaxic coordinates: hard cover edition, Paxinos, George, and Keith B.J. Franklin, page 60, Copyright Elsevier (2001)) at the same rostro-caudal location as the example slice.

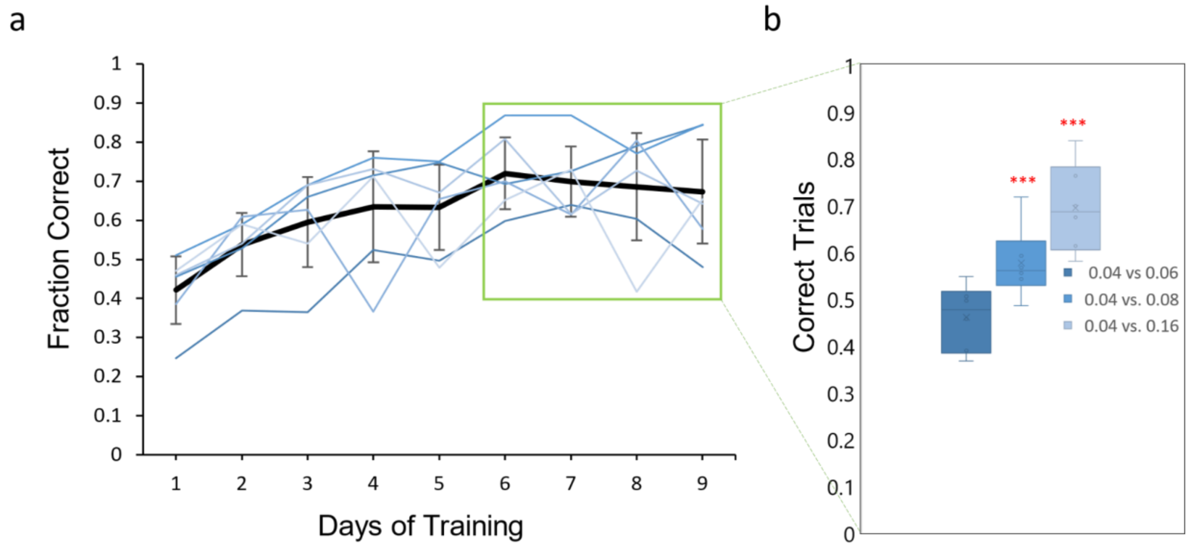

Figure S15. Learning curves and central SF discrimination.

(**a**) Learning curves from all six mice in the SF discrimination protocol. The target SF was 0.16 cpd, and the non-target distractor stimulus was set to 0.04 cpd. All stimuli had a low SF bandwidth (0.004 cpd) and a low orientation bandwidth (5°). The thick black line indicates the group mean. Error bars show the standard deviation of the mean.
(**b**) Average discrimination performances of all mice for different target-distractor combinations. After the mice reached expert performance (last for sessions in panel a, green square), we tested different SFs for the target stimulus to obtain an estimate of the animals' SF discrimination threshold. Discrimination performance quickly degraded when reducing the target-distractor SF difference, suggesting that the 0.04 versus 0.16 target-distractor difference was already close to their discrimination threshold. Correct trials for '0.04 vs 0.06 cpd' = 0.46 ± 0.02, T-test against 0.5, $p = 5.9 \times 10^{-2}$; '0.04 vs 0.08 cpd' = 0.58 ± 0.02, $p = 1.4 \times 10^{-4}$; '0.04 vs 0.16 cpd' = 0.69 ± 0.02, $p = 1.4 \times 10^{-8}$, mean ± s.e.m, n = 6 mice. Significance threshold was $\alpha = 0.0167$ after Bonferroni correction for performing three tests. Box plots indicate the median (horizonal line), interquartile range (box bounds: 25th–75th percentiles), and whiskers (1.5× interquartile range).

References

1. Mathis, A., Mamidanna, P., Cury, K.M. et al. DeepLabCut: markerless pose estimation of user-defined body parts with deep learning. Nat Neurosci 21, 1281–1289 (2018).

2. Portilla, J. & Simoncelli, E. P. A Parametric Texture Model Based on Joint Statistics of Complex Wavelet Coefficients. *Int. J. Comput. Vis.* **40**, 49–70 (2000).

3. Wang, Q. et al. The Allen Mouse Brain Common Coordinate Framework: A 3D Reference Atlas. Cell 181, 936-953.e20 (2020).
