# Supplementary material for: Broadband visual stimuli improve neuronal representation and sensory perception
Source: Nat Commun. 2025 Mar 26;16:2957. doi: 10.1038/s41467-025-58003-1 (PMC11947450; doi:10.1038/s41467-025-58003-1)
Supplement: Supplementary file 2 — Description of Additional Supplementary Files [file 41467_2025_58003_MOESM2_ESM.pdf]

Description of Additional Supplementary Files

File name: Supplementary Movie 1
Description: Example movie of a full-frame, narrow orientation bandwidth and narrow spatial frequency bandwidth stimulus (labeled in the manuscript as “narrow”).

File name: Supplementary Movie 2
Description: Example movie of a full-frame, narrow orientation bandwidth and broad spatial frequency bandwidth stimulus (labeled in the manuscript as “SF”).

File name: Supplementary Movie 3
Description: Example movie of a full-frame, broad orientation bandwidth and narrow spatial frequency bandwidth stimulus (labeled in the manuscript as “ORI”).

File name: Supplementary Movie 4
Description: Example movie of a full-frame, broad orientation bandwidth and broad spatial frequency bandwidth stimulus (labeled in the manuscript as “mixed”).

File name: Supplementary Movie 5
Description: Example of a Touchscreen chamber experimental trial with captioned timeline.
